# The molecular mechanisms of fluoroquinolone resistance found in rectal swab isolates of Enterobacterales from men undergoing a transrectal prostate biopsy: the rationale for targeted prophylaxis

**DOI:** 10.1186/s12941-021-00487-y

**Published:** 2021-12-07

**Authors:** Katarzyna Piekarska, Katarzyna Zacharczuk, Tomasz Wołkowicz, Mateusz Mokrzyś, Natalia Wolaniuk, Magdalena Nowakowska, Stanisław Szempliński, Jakub Dobruch, Rafał Gierczyński

**Affiliations:** 1 Department of Bacteriology, National Institute of Public Health NIH – National Research Institute, Chocimska 24, 00-791 Warsaw, Poland; 2 Department of Urology, St. Anna Hospital, A. Mickiewicza 39, 05-500 Piaseczno, Poland; 3 Department of Urology, Centre of Postgraduate Medical Education, 01-813 Warsaw, Poland

**Keywords:** Prostate biopsy, Post-biopsy infections, *Enterobacteriaceae*, Fluoroquinolone resistance, PMQR and mutations

## Abstract

**Background:**

Transrectal ultrasound-guided prostate biopsy (TRUS-Bx) is considered an essential urological procedure for the histological diagnosis of prostate cancer. It is, however, considered a “contaminated” procedure which may lead to infectious complications. Recent studies suggest a significant share of fluoroquinolone-resistant rectal flora in post-biopsy infections.

**Methods:**

The molecular mechanisms of fluoroquinolone resistance, including PMQR (plasmid-mediated quinolone resistance) as well as mutation in the QRDRs (quinolone-resistance determining regions) of *gyrA*, *gyrB*, *parC* and *parE*, among Enterobacterales isolated from 32 of 48 men undergoing a prostate biopsy between November 2015 and April 2016 were investigated. Before the TRUS-Bx procedure, all the patients received an oral antibiotic containing fluoroquinolones.

**Results:**

In total, 41 Enterobacterales isolates were obtained from rectal swabs. The MIC of ciprofloxacin and the presence of common PMQR determinants were investigated in all the isolates. Nine (21.9%) isolates carried PMQR with *qnrS* as the only PMQR agent detected. DNA sequencing of the QRDRs in 18 Enterobacterales (*E. coli* n = 17 and *E. cloacae* n = 1) isolates with ciprofloxacin MIC ≥ 0.25 mg/l were performed. Substitutions in the following codons were found: GyrA—83 [Ser → Leu, Phe] and 87 [Asp → Asn]; GyrB codon—605 [Met → Leu], ParC codons—80 [Ser → Ile, Arg] and 84 [Glu → Gly, Met, Val, Lys], ParE codons—458 [Ser → Ala], 461 [Glu → Ala] and 512 [Ala → Thr]. Six isolates with ciprofloxacin MIC ≥ 2 mg/l had at least one mutation in GyrA together with *qnrS*.

**Conclusions:**

This study provides information on the common presence of PMQRs among Enterobacterales isolates with ciprofloxacin MIC ≥ 0.25 mg/l, obtained from men undergoing TRUS-Bx. This fact may partially explain why some men develop post-TRUS-Bx infections despite ciprofloxacin prophylaxis.

## Introduction

Prostate cancer is one of the most prevalent and frequently diagnosed cancer in men in industrialized countries and is currently one of the most important health concerns [1, 2]. Transrectal ultrasound-guided prostate biopsy (TRUS-Bx) is a primary procedure to verify a prostate cancer diagnosis, frequently performed in urology [3]. TRUS-Bx is considered a “contaminated” procedure that may trigger infectious complications including urinary tract infection, acute bacterial prostatitis, epididymitis, orchitis and, most importantly, sepsis [3–6]. It is considered that pre-biopsy antimicrobial prophylaxis is effective in reducing post-TRUS-Bx infections [7, 8] and clinical guidelines therefore recommended antibiotic prophylaxis administered before the procedure as a standard of care to protect against bacterial complications [9–11]. Fluoroquinolones (FQs) are the most commonly used prophylaxis agents in urological procedures recommended by numerous international urology associations [12–15]. However, fluoroquinolone resistance among *Enterobacteriaceae* is increasingly more often reported worldwide. Moreover, the presence of FQ resistant bacteria within the rectum of patients undergoing a biopsy are considered an important risk factor for complications [5, 10, 13], with FQ-resistant *E. coli* being the most common cause of post-biopsy complications [5–7, 10, 16, 17].

The major FQ resistance mechanism is associated with mutations in the quinolone resistance determining region (QRDR) of chromosomal genes encoding DNA gyrase subunits (GyrA, GyrB) and topoisomerase IV (ParC, ParE) or their accumulation as has been previously reported [18–20]. Additionally, FQ resistance can also be caused by the co-existence of the aforementioned mutations and plasmid-mediated quinolone resistance (PMQR) determinants [20, 21]. Although PMQRs, including the Qnr proteins, a variant of aminoglycoside acetyltransferase—AAC(6')-Ib-cr, the QepA and OqxAB efflux pumps are considered factors providing only low-level resistance, their presence may stimulate mutations in the DNA gyrase and/or topoisomerase IV genes, resulting in high-level resistance to FQs [22].

In Europe, resistance to FQs is widespread. Antimicrobial resistance surveillance data from the European Centre for Disease Prevention and Control (ECDC) [https://www.ecdc.europa.eu/en/antimicrobial-resistance/surveillance-and-disease-data/data-ecdc] indicate high percentage in resistance to FQs among invasive Enterobacteriaceae isolates in certain countries, which is a cause for concern. According to these data, nearly 30% of *E. coli* and over 60% of *K. pneumoniae* isolates collected in Poland in the past years were resistant to FQs. To date, two reports from Poland has shown that ciprofloxacin resistant *E. coli* isolates were obtained among 9.6% (10/104) and 50.9% (57/112) of patients undergoing TRUS-Bx [23, 24]. However, there are no data about the level of ciprofloxacin resistance (MICs range) and the molecular mechanisms of FQ resistance in this patient group. Therefore, the objective of this study was to investigate the molecular mechanisms of FQ resistance in Enterobacterales isolates obtained from patients undergoing a prostate biopsy in Poland.

## Materials and methods

### Patients

This prospective study was conducted between November 2015 and April 2016 at a Urological Department in Warsaw, Poland. The study was approved by the local bioethics committee at the Centre of Postgraduate Medical Education (Research Bioethics Committee No. 39/PB/2014), Warsaw, Poland. An informed consent was obtained from all recruited patients prior to taking the swab. A total of 48 men suspected of prostate cancer and undergoing TRUS-Bx were enrolled in the study. In all the patients, antibiotic prophylaxis was used (ciprofloxacin [2 × 500 mg], levofloxacin [1 × 500 mg], norfloxacin [2 × 400 mg] or fosfomycin trometamol [1 × 3 g]), starting two hours to 2 days before TRUS-Bx (with two exceptions where it started 5 and 6 days beforehand).

### Bacterial isolates and ciprofloxacin susceptibility testing

In all the 48 patients, rectal swabs were collected by using a cotton swab with a transport system and, within a short time, the swabs were transported to a microbiological laboratory. Subsequently, according to the methodology described previously [23, 25] and our standard laboratory procedures, all the rectal swabs were streaked onto Columbia Agar with 5% sheep blood (bioMerieux, France) and the McConkey (Becton Dickinson Poland) medium. The plates were incubated aerobically 18–24 h at 37° C. The species identification of cultured bacteria was performed by using classic biochemical methods. For all the collected isolates, the minimal inhibitory concentration (MICs) of ciprofloxacin was determined by using the E-test method. MIC results were interpreted according to the European Committee on Antimicrobial Susceptibility Testing (EUCAST) criteria (http://www.eucast.org/clinical.breakpoints/) and MIC values > 0.5 mg/l were classified as resistance to FQs. The MIC for each isolate was measured at least twice.

### PCR and DNA sequencing

All the isolates obtained were screened for the presence of PMQR determinants (*qnrA*, *qnrB*, *qnrC*, *qnrD*, *qnrS*, *aac(6')-Ib* and *qepA*) by means of PCR using primers and reaction conditions as previously described [20, 26]. All of the PCR products for PMQR genes were confirmed by direct Sanger DNA sequencing. Additionally, from all of the isolates that were PMQR-positive and/or resistant to ciprofloxacin, PCR and DNA sequencing of the QRDRs of *gyrA*, *gyrB*, *parC* and *parE* genes were performed by using primers described previously, for *gyrA* and *parC* [27], *gyrB* and *parE* [18], respectively.

### Genetic relatedness

The genetic relatedness of FQ-resistant and/or PMQR-positive *E. coli* isolates was analysed by pulsed-field gel electrophoresis (PFGE) as previously described [20]. PFGE was conducted using the CHEF-DR II system (Bio-Rad Laboratories, USA) and the *Xba*I endonuclease (EURx, Poland). PFGE-patterns were analysed using BioNumerics software v6.6. (Applied Maths, Sint-Martens-Latem, Belgium). Similarity clustering analyses were performed using UPGMA and Dice correlation coefficient with a tolerance of 1.2%.

## Results

### Patients

A total of 48 patients undergoing a prostate biopsy between November 2015 and April 2016 participated in this study. The patients’ median age was 70.46 (range 49–89). All the patients received an oral antibiotic, usually ciprofloxacin (41/48). Other antibiotics, including levofloxacin, norfloxacin or fosfomycin trometamol, were used in 5, 1 and 1 patient, respectively. Furthermore, a different number of ciprofloxacin doses administered as prophylaxis was recorded (Table 2).

### Isolates and their sensitivity to ciprofloxacin

A total of 41 *Enterobacterales* isolates were obtained from the rectal swabs from 32 men undergoing a transrectal prostate biopsy. The collected isolates were identified as *E. coli* (n = 38), *E. cloaceae* (n = 2) and *P. vulgaris* (n = 1). Because of the lack of sufficient medical evidence to consider *E. cloaceae* or *P. vulgaris* aetiological agents of post biopsy infection, cultured ciprofloxacin-sensitive isolates of these two species were excluded from further investigations. The only exception was ciprofloxacin resistant *E. cloacae* isolate 10 II (Table 2). All the isolates showed ciprofloxacin MICs values from 0.008 mg/l to ≥ 32 mg/L (Table 1). Ciprofloxacin-resistant isolates (13/38 *E. coli* and 1/2 *E. cloacae*) with MIC > 0.5 mg/l according to the EUCAST criteria were detected in 14 (29.2%) out of 48 TRUS-Bx patients (Table 2). From one patient, *E. coli* (isolate No. 10 I) with MIC ciprofloxacin 0.25 mg/L and *E. cloacae* (isolate No. 10 II) with MIC ciprofloxacin 2 mg/L were detected (Table 2). Furthermore, from the rectal swabs of 3 (6.25%) patients, 3 distinct *E. coli* isolates with a MIC range of 0.25–0.5 mg/L were isolated. All the patients with resistant isolates or isolates with decreased susceptibility to ciprofloxacin (MIC range 0.25–0.5 mg/L) received ciprofloxacin before the biopsy in a different number of doses—from 1 to 12.

**Table 1:** Distribution of ciprofloxacin minimum inhibitory concentration (MICs) in 41 Enterobacterales strains isolated from 48 men suspected of prostate cancer and undergoing transrectal ultrasound-guided prostate biopsy

Strain	MIC (mg/l) of CIP:																								
	0.008	0.012	0.016	0.023	0.032	0.047	0.064	0.094	0.125	0.19	0.25	0.38	0.5	0.75	1.0	1.5	2	3	4	6	8	12	16	24	≥ 32
*E. coli* (n = 38)	1	4	6	7	–	2	–	–	–	1	3	–	1	–	–	–	–	–	–	1	3	2	–	2	5
*E. cloacae* (n = 2)	–	–	–	–	1	–	–	–	–	–	–	–	–	–	–	–	1	–	–	–	–	–	–	–	–
*P. vulgaris* (n = 1)	–	–	–	–	–	–	–	1	–	–	–	–	–	–	–	–	–	–	–	–	–	–	–	–	–

*CIP* ciprofloxacin

**Table 2:** Ciprofloxacin number of doses used, minimum inhibitory concentration (MICs) and mechanisms of fluoroquinolone resistance detected among 18 Enterobacteriaceae strains isolated from 16 men undergoing transrectal ultrasound-guided prostate biopsy

Isolate no	Species	Date of isolation	Antibiotic-course treatment and number of doses	MIC CIP (mg/L)	Mutation(s)								PMQRs
					GyrA		GyrB	ParC		ParE			
9 I	*E. coli*	4.12.2015	1 × Cip	0.25	–	–	M605L	–	–	–	–	–	*qnrS*1
10 I	*E. coli*	11.12.2015	1 × Cip	0.25	S83L	–	–	–	–	–	–	–	*qnrS*1
40	*E. coli*	25.03.2016	1 × Cip	0.25	–	–	–	–	–	–	V461E	–	*qnrS*1
41	*E. coli*	25.03.2016	1 × Cip	0.5	S83L	–	–	S80R	–	–	–	–	–
10 II	*E. cloaceae*	11.12.2015	1 × Cip	2	S83F	–	–	–	–	–	–	A512T	*qnrS*1
6	*E. coli*	4.12.2015	5 × Cip	6	S83L	D87N	–	S80I	–	–	–	–	–
11	*E. coli*	11.12.2015	3 × Cip	8	S83L	D87N	–	S80I	–	–	–	–	–
14	*E. coli*	15.01.2016	5 × Cip	8	S83L	–	–	–	E84G	–	–	–	*qnrS*1
42	*E. coli*	25.03.2016	2 × Cip	8	S83L	D87N	–	S80I	–	–	–	–	–
18 I	*E. coli*	23.01.2016	1 × Cip	12	S83L	D87N	–	S80I	–	–	–	–	–
34 I	*E. coli*	11.03.2016	12 × Cip	12	S83L	D87N	–	S80I	–	–	–	–	*qnrS*
20	*E. coli*	5.02.2016	1 × Cip	24	S83L	D87N	–	S80I	–	–	–	–	–
38	*E. coli*	18.03.2016	2 × Cip	24	S83L	D87N	–	S80I	–	–	–	–	–
33	*E. coli*	11.03.2016	4 × Cip	32	S83L	D87N	–	S80I	–	–	–	–	*qnrS*1
12	*E. coli*	11.12.2015	3 × Cip	> 32	S83L	D87N	–	S80I	E84M	–	–	–	–
28	*E. coli*	4.03.2016	3 × Cip	> 32	S83L	D87N	–	S80I	E84V	–	–	–	*qnrS*
34 II	*E. coli*	11.03.2016	12 × Cip	> 32	S83L	D87N	–	S80I	–	S458A	–	–	–
43	*E. coli*	25.03.2016	10 × Cip	> 32	S83L	D87N	–	S80R	E84K	–	–	–	*qnrS*2

*Cip* ciprofloxacin

### The presence of mutations in the quinolone resistance-determining regions (QRDRS) of *gyrA*, *gyrB*, *parC*, *parE* and plasmid-mediated quinolone resistance determinants

DNA sequencing of the QRDRs in *gyrA*, *gyrB, parC* and *parE* showed that all of the 18 *Enterobacteriaceae* isolates with ciprofloxacin MIC range ≥ 0.25 mg/L had a point mutation that involved at least one amino acid substitution. The substitutions were observed in two GyrA codons: 83 [Ser → Leu(n = 15), Phe (n = 1)] and 87 [Asp → Asn (n = 12)]; one GyrB codon: 605 [Met → Leu (n = 1)]; two ParC codons: 80 [Ser → Ile (n = 11), Arg (n = 2)] and 84 [Glu → Gly, Met, Val, Lys]; three ParE codons: 458 [Ser → Ala (n = 1)], 461 [Glu → Ala (n = 1)] and 512 [Ala → Thr (n = 1)], respectively. The majority of the isolates with ciprofloxacin MICs ≥ 6 mg/L (12/18; 66%) had double mutations in GyrA (Ser83 → Leu and Asp87⟶Asn) and at least one mutation at codon ParC80 (11 isolates had substitution Ser → Ile and one isolate had substitution Ser → Arg). Moreover, three out of four *E. coli* isolates (except No. 34 II) with a MIC of > 32 mg/L for ciprofloxacin had an additional alteration at codon Glu84 in *parC* QRDR (Glu84 → Met, Val, Lys) (Table 2). In isolate No. 14 with a ciprofloxacin MIC of 8 mg/L, substitutions at codon 84 of ParC (Glu → Gly) and at codon 83 of GyrA (Ser → Leu) were detected. Three isolates (No. 9 I, 10 I and 40) with ciprofloxacin MICs of 0.25 mg/L carried only one amino acid substitution in GyrA or ParE, respectively (Table 2). Among *E.coli* isolates with ciprofloxacin MICs of ≥ 0.5 mg/L, only one type of amino acid substitution at codon 83 (Ser83 → Leu) in GyrA was observed, whereas ciprofloxacin-resistant *E. cloacae* isolate (isolate No. 10 II) did have alterations at this codon (Ser83 → Phe).

In total, PMQR determinants were present among 9 (21.9%) out of 41 *Enterobacterales* isolates obtained from the rectal swabs in this study. Half (9/18) of the tested isolates with MICs ciprofloxacin ranging from 0.25 to ≥ 32 mg/L carried PMQR. The *qnrS* was the only PMQR determinant detected in this study. Six isolates had a *qnrS1* variant, one—a *qnrS2* variant and two isolates had a *qnrS*–like gene. What is more, the *qnrS* gene was detected in 6 out of 14 ciprofloxacin-resistant isolates (Table 2). In contrast, other PMQR genes investigated in this study (*qnrA*, *qnrB*, *qnrC*, *qnrD*, *aac(6')-Ib, qepA* and *oqx*AB) were not detected in any of the isolates tested.

### Pulsed-field gel electrophoresis typing analysis

**Fig. 1:**
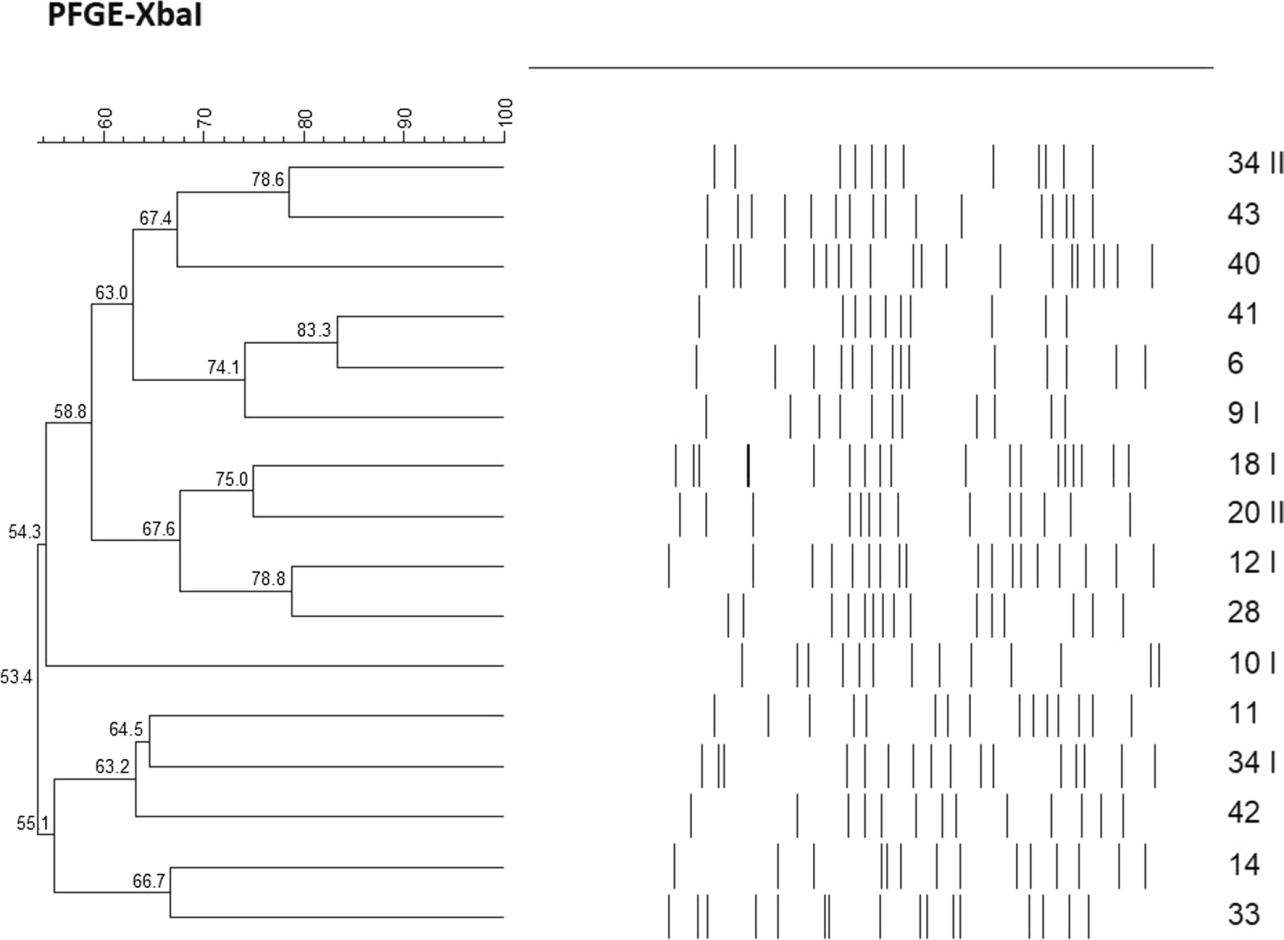
Dendogram of PFGE-XbaI profiles of 16 fluoroquinolone-resistant pre–prostate biopsy rectal *Escherichia coli* isolates. Analysis was made with BioNumerics software v6.6 (Applied Maths, Belgium) by th UPGMA alghoritm based on Dice similarity coefficients (optimization, 1.5%; position tolerance, 1.5%)

According to PFGE analysis, the similarity of Xba-PFGE profiles obtained for 16 *E.coli* isolates ranged from 53.4% to 83.3%. One *E. coli* isolate (No. 38) was untypable by PFGE (Fig. 1). The PFGE typed *E. coli* isolates were non clonal.

## Discussion

According to the European Association of Urology (EAU), there is a strong recommendation to use antimicrobial prophylaxis in men prior to a transrectal prostate biopsy (TRUS-Bx) in order to minimise the risk of bacterial infections after the procedure [15]. Suggested regimens for antimicrobial prophylaxis included fluoroquinolones and cephalosporins, fosfomycin or aminoglycosides if FQ resistance is present [15]. In fact, as EAU experts underline, a specific antibiotic should be chosen by the urologist based on the local pathogen profile and antibiotic susceptibility patterns and virulence [15].

Fluoroquinolones (FQ), especially ciprofloxacin, are widely used as prophylaxis for TRUS-Bx in many countries as well as constitute frequently prescribed antibiotics for treating urinary tract infections (UTIs) in men, such as bacterial prostatitis and epididymitis [11, 15]. However, the high overuse of fluoroquinolones in past decades has resulted in an increasing rate of bacterial isolates resistant to these agents and can have an important bearing on the success or failure of prophylaxis.

It is recognized that the rectal flora is the source for most UTI infections, including those occurring after TRUS-Bx [28]. Moreover, isolates of *E.coli*, which is the part of human intestinal flora, are most commonly associated with post-TRUS-Bx infections [8, 11, 17, 29]. Several studies [8, 23, 24, 29–31] showed FQ-resistant *E. coli* as reservoir infections following a prostate TRUS-Bx. The aforementioned studies present varying rates from 9.2% to 50.9% of FQ-resistant *E coli* detected. These two extreme rates were observed in studies conducted in Poland [23, 24]. In this study, 29.2% prevalence of ciprofloxacin-resistant *Enterobacterales* isolates (13 *E. coli* and 1 *E. cloacae*) was found in the pre-TRUS-Bx rectal swab samples of the study population, which constituted 34% of all the isolates obtained from the rectal swabs tested.

Our previous studies [20] found that FQ resistance is often associated with the accumulation of different mechanisms within one resistant clinical isolate. Therefore, we broadly characterised the molecular FQ resistance mechanisms including mutations in the QRDRs of genes (*gyrA*, *gyrB* and *parC*, *parE*) subunits and PMQRs (*qnrA*, *qnrB*, *qnrS*, *qnrC*, *qnrD*, *aac(6')-Ib, qepA* and *oqx*AB) in isolates from patients undergoing a prostate biopsy, in Poland. To the best of our knowledge, the study presented herein is the first report on the molecular mechanisms of FQ resistance in this patient group in Poland.

As it is well known, clinically relevant fluoroquinolone resistance is most commonly affected by point mutations in the genes coding DNA gyrase and topoisomerase IV and play an essential role in quinolone resistance [19, 20, 27, 32]. Similarly to other reports, this study found that the most common mutations were at 83 and 87 amino acid position of GyrA, and at 80 and 84 amino acid position of ParC (Table 2) [20, 30, 32]. All the 14 isolates resistant to ciprofloxacin (MIC > 0.5 mg/l) found in this study had a mutation at Ser-83 in GyrA QRDR, supporting the hypothesis that an alteration of a single amino acid at this codon is sufficient to decrease susceptibility to ciprofloxacin, as previously noted [20]. Moreover, this hypothesis is also confirmed by two detected isolates of *E. coli* with ciprofloxacin MICs 0.25–0.5 mg/L and mutations at codon 83 GyrA. In fact, the mutations could have serious implications to the development of infections occurring after TRUS-Bx, even after a single dose of FQ. Besides, in 12 ciprofloxacin resistant *E. coli* isolates with MIC > 6 mg/L, alterations at codon 87 GyrA and at codon 80 ParC were observed. Similar mutations were reported in the USA where 13 *E. coli* isolates with ciprofloxacin MIC ≥ 4 mg/L obtained from patients with serious infections occurring after a prostate biopsy had alterations at codons 83 (Ser → Leu) and 87 (Asp → Asn) of GyrA and at codon 80 (Ser → Ile) of ParC [30]. This phenomenon of accumulated simultaneous alterations in GyrA (codons 83 and 87) and ParC (codons 80 and 84) subunits promote the development of high-level resistance (MIC > 32 mg/L) to FQs. In this study, 3 *E. coli* isolates with ciprofloxacin MIC > 32 mg/L and the aforementioned mutations were found (Table 2). Furthermore, in this study, the majority of *E. coli* isolates with ciprofloxacin MIC > 6 mg/L had three (n = 9) or four (n = 3) point mutations in GyrA and ParC encoding for an amino acid substitution. Our results may suggest a correlation between the accumulation of mutations in GyrA and ParC subunits and high-level FQ resistance. It is worth noting that isolate No. 41 with a ciprofloxacin MIC of 0.5 mg/L detected in our study had alterations in GyrA (Ser83 → Leu) and ParC (Ser8 → Ile) typical of resistant isolates.

PMQR determinants are considered to play an important role in the acquisition of high-level FQ resistance by the selection of mutations in QRDRs gyrase and topoisomerase IV, mainly in *gyrA* and *parC* [32]. Little is known on PMQRs in *Enterobacterales* isolated from men undergoing a prostate biopsy and receiving ciprofloxacin prophylaxis. Significant prevalence (18.75%) of PMQR determinants among *Enterobacterales* isolates from TRUS-Bx patients is reported herein. Nine (n = 8 *E. coli* and n = 1 *E. cloacae*) out of the 18 isolates detected with ciprofloxacin MICs of ≥ 0.25 mg/L carried a PMQR determinant, *qnrS* gene. Interestingly, other PMQRs (*qnrA*, *qnrB*, *qnrC*, *qnrD*, *aac(6')-Ib* and *qepA*) were not found, despite the tested isolates being not clonal. In this study, two *E. coli* (No. 9 I and 40) isolates (ciprofloxacin MIC 0.25 mg/L) with *qnrS* and no alterations in GyrA and ParC were identified. This may support the hypothesis that PMQR determinants promote mutations in QRDR. The third *E. coli* isolate with a ciprofloxacin MIC of 0.25 mg/L and *qnrS* had an amino acid alteration at codon 83 of gyrase A, which is considered a primary target for FQs [32]. It is worth noting that the majority of QnrS-positive isolates found in our study with a ciprofloxacin MIC range of 0.25 to > 32 mg/L had from 1 to 4 mutations in QRDRs. Because PFGE typing of *E. coli* isolates revealed no clonality the occurrence of QnrS-positive isolates may be common.

Notably, the aforementioned FQ resistance traits found in *Enterobacterales* isolates from patients receiving ciprofloxacin prophylaxis prior to TRUS-Bx were of the same type as the one commonly reported for isolates collected from patients receiving regular FQ treatment. This finding may indicate that FQ prophylaxis may be ineffective in patients who carry FQ-resistant isolates due to a prior FQ therapy or isolates with decreased FQ sensitivity due to foodborne or environmental exposition to low FQ concentrations [33–36]. It should be noted that, in Poland, *E. coli* and *Salmonella* isolates with the same as in this study amino acid alterations at codons Ser83 and Asp87 of GyrA and at codon Ser80 of ParC and *qnrS*1 as the most common PMQRs were identified from animals or retail food [33–35]. This finding may suggest that the consumption of food contaminated with PMQR-producing isolates as well as a contact with animals hosting such isolates may be related to FQ resistance detected in humans. Consideration of an alternative to FQ antibiotic prophylaxis would be reasonable. In the light of our findings, urologists should be aware of the increased risk of FQ resistance in bacteria and consider microbiological diagnostic measures, such as rectal swab cultures, to determine antibiotic susceptibility before a prostate biopsy. This is in line with the conclusions and recommendations of other authors [37, 38]. Additionally, our data may also indicate the need for molecular PMQRs testing in isolates with reduced ciprofloxacin susceptibility. A recent study by Lee et al*.* suggests that targeted prophylaxis may be cost-effective [38].

## Limitations

The limitations of our study include the relatively low number of investigated patients and, consequently, the low number of isolates cultured. Furthermore, patients from a single healthcare facility were tested.

## Conclusions

This study provides information on the common presence of PMQRs among Enterobacterales isolates with ciprofloxacin MIC ≥ 0.25 mg/l, obtained from men undergoing TRUS-Bx. This fact may partially explain why some men develop post-TRUS-Bx infections despite ciprofloxacin prophylaxis.

## Data Availability

All the datasets used and/or analysed in the presented study are available at the corresponding author upon reasonable request. The authors have confirmed that personal identity information of the patient data was unidentifiable in this report.

## Abbreviations

TRUS-Bx: Transrectal ultrasound-guided prostate biopsy
FQ: Fluoroquinolones
QRDRs: Quinolone-resistance determining regions
*gyrA* and *gyrB*: Chromosomal genes encoding GyrA and GyrB subunits of DNA gyrase
*parC* and *parE*: Chromosomal genes encoding ParC and ParE subunits of topoisomerase IV
PMQR: Plasmid-mediated quinolone resistance
ECDC: The European Centre for Disease Prevention and Control
MIC: Minimal inhibitory concentration
EUCAST: The European Committee on Antimicrobial Susceptibility Testing
PCR: Polymerase chain reaction
PFGE: Pulsed-field gel electrophoresis
EAU: The European Association of Urology
UTI: Urinary tract infections
CIP: Ciprofloxacin
